# The Importance of the Discourse on the Method

**DOI:** 10.1186/1753-2000-4-2

**Published:** 2010-01-07

**Authors:** Benedetto Vitiello

**Affiliations:** 1 Child and Adolescent Treatment and Preventive Intervention Research Branch, National Institute of Mental Health, Bethesda, Maryland, USA

## 

Central to the research enterprise is the application of scientific methods appropriate to achieving the investigational aims. In the case of controlled clinical trials, assay sensitivity is achieved through careful selection of study sample, design, and assessment instruments. Treatments and controls must be precisely defined and quantified. Concomitant interventions need to be specified and delimited. The actual implementation of the protocol requires procedures for ensuring fidelity, consistency, and reproducibility. Typically, a clinical trial protocol is the result of contributions from experts in clinical care, experimental methodology, bioethics, statistics, and data management.

Appreciating the research methodology of a study is critical for understanding the value and the limitations of the results. The quality of methods is especially important in the case of so called "negative" trials, as are often called studies that, though designed to reject a null hypothesis of equivalence, do not find significant differences between treatments. Distinguishing a truly negative trial (i.e., a study indicating that no difference between treatments actually exists) from a "failed trial" (i.e., a study that lacks assay sensitivity and is unable to detect a difference even if it exists) depends primarily on the strength of the research methodology of the trial.

While considerable effort usually goes into preparing the methods for a controlled clinical trial, only a fraction of this work is actually mentioned in the publication of the results. There are several reasons for this situation. First, space limitations prevent a detailed description of the methodology and a critical discussion of the theoretical and practical reasons for the choices that were made vis-à-vis the possible alternatives. Second, though the interpretation of the results hinges on a thorough understanding of the methods that were used, many readers of scientific literature would not have the time of entering into these details. Lastly, in order to appreciate the methodological structure behind a research study, one needs to have some basic expertise in it, something that not all the consumers of clinical research actually have.

However, a more detailed sharing of clinical trial methods than is commonly done in the typical publication of the study results is valuable from a number of perspectives. Researchers reading their colleagues' work can have the opportunity of appreciating the elements of the study and understanding the rationale for choosing a particular design and specific measurements. Even if all clinical trials share a common experimental basis, there can be also considerable innovation underway, at the level of design, sample selection, measurement tools, bioethical assessment, and statistical analyses [1,2]. It is fair to say that, regardless of any standardization of design, treatments and assessment tools, many clinical trials have unique features regarding their methods and/or their implementation. More detailed descriptions of study methods and critical discussions of their strengths and limitations can foster the development of more sensitive, valid, efficient, and ultimately informative clinical research. Finally, publishing the study methodology separately and in advance of the results papers offers the authors the practical benefit of being able to refer the readers to a detailed description of the study background when reporting the results.

In recent years, a movement towards publishing design and methods considerations separately from the result reports has occurred both in adult and child psychiatry [3-6]. The report by Compton et al., published in CAPMH this month, outlines the rationale, design, and methods of the Child/Adolescent Anxiety Multimodal Study (CAMS) [7], and provides another example of how this type of papers can help us better understand and appreciate the results of clinical trials. Along these lines, CAPMH welcomes the submission of similar manuscripts describing and discussing the methodological aspects of clinical trials in child and adolescent mental health.
